# From Melanocyte to Metastatic Malignant Melanoma

**DOI:** 10.1155/2010/583748

**Published:** 2010-08-11

**Authors:** Bizhan Bandarchi, Linglei Ma, Roya Navab, Arun Seth, Golnar Rasty

**Affiliations:** ^1^Department of Applied Molecular Oncology, Princess Margaret Hospital, Ontario Cancer Institute, University of Toronto, 7 Yorkview Drive, Toronto, ON, Canada M2N 2R9; ^2^Department of Pathology, University of Michigan Hospital, University of Michigan, Michigan 48109-0602, USA; ^3^Department of Laboratory Medicine and Pathobiology, University of Toronto, Toronto, Canada M4N 3M5; ^4^Department of Laboratory Medicine and Pathobiology, University Health Network, University of Toronto, Toronto, Canada M5G 2C4

## Abstract

Malignant melanoma is one of the most aggressive malignancies in human and is responsible for almost 60% of lethal skin tumors. Its incidence has been increasing in white population in the past two decades. There is a complex interaction of environmental (exogenous) and endogenous, including genetic, risk factors in developing malignant melanoma. 8–12% of familial melanomas occur in a familial setting related to mutation of the CDKN2A gene that encodes p16. The aim of this is to briefly review the microanatomy and physiology of the melanocytes, epidemiology, risk factors, clinical presentation, historical classification and histopathology and, more in details, the most recent discoveries in biology and genetics of malignant melanoma. At the end, the final version of 2009 AJCC malignant melanoma staging and classification is presented.

## 1. Introduction

The epidermis contains two types of dendritic cells, besides keratinocytes, that could mimic each other: Langerhans' cells and melanocytes. Langerhans' cells are dendritic antigen processing cells and hence play a primary role in cellular response to tumor antigens, skin graft rejection, and microorganism [1, 2]. Langerhans' cells are located in the suprabasal layer of the epidermis, a feature differentiating them from melanocytes on routine H&E stain. Melanocytes originate from the neural crest and in contrast to Langerhans' cells are located amongst the basal layer of the epidermis, hair bulb, eyes, ears, and meninges [3–5]. Melanocyte migration to the epidermis, function, and its survival are all dependant on expression of the tyrosine kinase receptor c-kit gene [4, 6]. The pigmentary system of the skin is a complex set of reactions with many potential sites for dysfunction [7]. Melanin pigment is produced by melanocytes in their specific cytoplasmic organelles called melanosomes. Melanosomes may represent a variant of lysosome [8], in which tyrosinase acts on the substrate tyrosine, resulting in dopa and dopaquinone formation [9, 10]. Melanin pigment synthesized by each melanocyte is transferred to an average of 36 keratinocytes. PAR-2 on the keratinocyte surface is a key receptor in this transfer [11, 12]. The transferred melanin then forms a cap at the top of nucleus of mitotically active basal cells and prevents the ultraviolet injurious effects on nucleus. Melanin stains with Masson-Fontana silver method, based on a positive argentaffin reaction, in which means the melanocytes take up silver and then reduce it to a visible metallic state, without the aid of a reducing agent.

## 2. Epidemiology

The incidence of malignant melanoma has been increasing in white populations [13–15]. Although malignant melanoma comprises less than 5% of malignant skin tumors; however, it is responsible for almost 60% of lethal skin neoplasia [16]. One of the highest incidence rates is in Queensland, Australia [17]. The incidence of malignant melanoma appears to be lower and stable in darkskin individuals (Africans, Native Americans, Asians, and Hispanics). Decreased incidence reported from some countries is probably partly due to an influx of low risk immigrants [18–21]. With increased life expectancy of the elderly population, melanoma will be a public health challenge [22]. Increased incidence of melanoma is partly due to early detection (thin melanomas) and partly due to true increase of incidence. Despite the increase in the incidence of melanoma, the prognosis has been improving due to earlier diagnosis of thin melanomas and hence in a curable stage [23–25]. The incidence of melanoma is equal in men and women and uncommon in children although there are reports that the incidence may be higher in women. A typical patient is usually a Caucasian adult in the 4th decade of life with lesion on the back and leg in male and female, respectively. One typical study revealed that the most common sites in decreasing order are the trunk (43.5%), extremities (33.9%), acral sites (11.9%), and head and neck (10.7%) [16].

## 3. Risk Factors

There is a complex interaction of environmental (exogenous) and endogenous factors. Up to 65% of malignant melanomas are sun-related [26–28]. The role of chronic sun exposure is controversial. Some studies suggested that total accumulated exposure to sun is a very important factor whereas long-term occupational exposure actually may be protective [29, 30]. In either case the general acceptance is that intermittent sun exposure is the most important factor. The list of risk factors in developing malignant melanoma is long and includes pale skin, blond or red hair, numerous freckles and tendency to burn and tan poorly (predominantly skin phototype 1–3) [26–28, 31], presence of more than 50 acquired (common, banal) nevi [32], more than five dysplastic (atypical, Clark's) nevi, large congenital nevi [33, 34], nevi larger than 6 mm [35], PUVA therapy, tendency to sunburn and tan poorly, use of tanning salons, Xeroderma pigmentosum, immunosuppression, chemical exposures, scars, Marjolin's ulcer [36–39], and genetic factors. In fact 8%–12% of malignant melanomas occur in a familial setting which may be related to mutations of the CDKN2A gene that encodes p16 and is linked to chromosome 9p21 [40, 41].

## 4. Clinical Presentation

Typical malignant melanomas usually present as “Malignant Melanoma ABCD”: asymmetry, border irregularity, color variegation, diameter more than 6 mm. However, many exceptions may occur as they may do in other medical disciplines. 

## 5. Diagnosis

Any suspicious pigmented lesions must be biopsied to rule out or rule in melanoma. Even though dermascopy, even in the hands of a relatively inexpert practitioner, may show high diagnostic accuracy [42] and boost the clinical suspicion in diagnosing malignant melanoma; however, the definitive diagnosis is confirmed done by biopsy.

## 6. Classification and Histopathology

Historically, malignant melanoma was classified by Wallace Clark and coworkers into superficial spreading type, lentigo malignant type, and nodular type [43, 44]. Later on Dr. Richard Reed added a fourth type called acral lentiginous malignant melanoma [45]. Since then the classification of malignant melanoma with their relative incidences has been as follows: superficial spreading melanoma (50%–75%), nodular melanoma (15%–35%), lentigo maligna melanoma (5%–15%), acral lentiginous melanoma (5%–10%), desmoplastic melanoma (uncommon), miscellaneous group (Rare). 

Melanoma presents three clinically and histomorphologically discernable steps in tumor progression [46].

1. Malignant Melanoma confined to the epidermis (melanoma in situ), which is called Radial Growth Phase- (RPG-) confined melanoma.
2. Radial Growth Phase (RGP)-confined microinvasive, which shows some malignant cells present in superficial papillary dermis.
3. Vertical Growth Phase (VGP), which means melanoma, has entered the tumorigenic and/or mitogenic phase (usually Clark's level II and occasionally Clark's level III).

The importance of RPG is best demonstrated by the Taran and Heenan study, which showed development of metastatic melanoma in only 5 of 1716 patients with level 2 melanomas (= 1 mm thick) in 7 to 14 years followup [47]. Those 5 cases with metastatic melanomas revealed regression. 

### 6.1. Superficial Spreading Melanoma (SSM)

SSM is the most common melanoma that can occur at any site and at any age [48]. About 75% of SSMs occur de novo. The classic lesions show variation in pigmentation and pagetoid spread of melanoma cell in epidermis.

### 6.2. Nodular Melanoma (NM)

NM melanoma by definition has no radial growth phase and could be nodular, polypoid, or pedunculated [49, 50].

### 6.3. Lentigo Maligna Melanoma (LMM)

This variant occurs on the sun-exposed skin, face, and upper extremities of elderly patients [51]. Lentigo maligna (also called Hutchinson freckle) is basically in situ melanoma and is characterized by epidermal atrophy, extensive solar, lentiginous, and back-to-back proliferation of melanoma cells with nest formation with extension into cutaneous adnexa. Only 5% of patients with lentigo maligna progress to lentigo maligna melanoma, and it usually takes several years [52]. Several methods of therapy can be used to treat lentigo maligna including cryotherapy, superficial radiation, and surgical excision with mapping and modified Mohs' surgery [53–55]. 

### 6.4. Acral Lentiginous Melanoma (ALM)

ALM is common on palmar, plantar, and ungual skin of Black and Japanese people [56]. Ulcerate and melanonychia striata may occur. Although this type of melanoma is common in the above-mentioned locations, other types of malignant melanoma may still develop at the same location [57]. Most of the mucosal melanomas including oral cavity, vulva vagina, and cervix uteri follow the histological features of acral lentiginous melanomas [58, 59].

## 7. Rare Variants

There are rare variants of malignant melanoma that do not show the typical classical histopathology. Amongst these variants, Desmoplastic Melanoma (DM)/Neurotropic Melanoma is worth mentioning more in detail since it could easily be misdiagnosed as fibroblastic proliferation and scar. This variant usually presents as indurated plaque or bulky tumor on the head and neck location [60] and is characterized by paucicellular proliferation of atypical dermal spindle melanocytes, dermal collections of lymphocytes with overlying epidermis commonly showing lentigo maligna [61]. This variant also commonly shows neurotropism. The dermal component of desmoplastic melanoma is usually negative for Melan A (or Mart1) and HMB45. These two immunostains, however, highlight the presence of Lentigo maligna in situ. Both the epidermal component and dermal atypical spindle melanocytes are positive for S100 immunostain. Other rare variants include nevoid melanoma [62, 63], verrucous melanoma, small cell melanoma, signet ring melanoma, myxoid melanoma, osteogenic melanoma of the finger, animal (Equine/pigment synthesizing) melanoma, childhood melanoma excongenital nevus, and minimal deviation malignant melanoma. Minimal deviation subtype is characterized by uniform proliferation of melanocytes that show minimal cytomorphological atypia [64].

## 8. Prognostic Factors in Melanoma

There are three classes of adverse prognostic factors in melanoma: pathological, clinical, and other factors including genetic alteration. The first group includes increasing the Breslow thickness [65], ulceration, mitotic rate, Clark level [44], absent or nonbrisk tumor infiltrating lymphocytes [66], regression [67, 68], microscopic satellites [69], lymphovascular invasion [70], angiotropism, tumor volume, neurotropism, cell type, local recurrence, histopathologic subtype, and presence of vertical growth phase. Clinical adverse factors include increasing age, male, location of the lesion, and metastasis.

## 9. The Biology and Genetics of Malignant Melanoma

Two genes have been discovered in melanoma families: CDKN2A (p16) on chromosome 9p21 and CDK4 on chromosome 12 [70, 71]. The CDKN2A gene acts as a tumor suppressor gene and plays a crucial role in cell cycle regulation and senescence. Mutations of the CDKN2A gene confer susceptibility to familial melanoma. Partial or complete loss of p16 expression has also been identified in sporadic melanomas. Other genes, such as MC1R (Melanocortin 1 Receptor) and DNA repair genes, are likely to be more important in determining susceptibility for melanoma in the general population [72]. 

Although nevi and melanomas share initiating genetic alterations such as oncogenic mutations in BRAF and NRAS, melanomas often show recurrent patterns of chromosomal aberrations such as losses of chromosomes 6q, 8p, 9p, and 10q along with gains of chromosomes 1q, 6p, 7, 8q, 17q, and 20q, while benign nevi tend to have no detectable chromosomal aberrations by comparative genomic hybridization (CGH) or karyotyping [73–75]. Recently, a fluorescence in situ hybridization- (FISH-) based test, using a combination of 4 FISH probes targeting 3 loci on chromosome 6 (RREB1 and MYB genes) and 1 on chromosome 11 (Cyclin D1 gene), was developed [76, 77]. The method is applicable to formalin-fixed paraffin-embedded tissue and has the most powerful discriminatory ability between melanoma and nevi. 

Metastatic melanoma is an incurable disease with high mortality rate. Patients with metastatic disease have an average survival of <1 year. This high mortality rate is largely the result of the resistance to chemotherapy and radiotherapy [78, 79]. Transformation of melanocytes to melanoma cells is still largely unclear [78]. A combination of up- or downregulation of various effectors acting on different molecular pathways appears to be involved in progression of normal melanocyte to metastatic malignant cells [80]. Numerous studies using tissue specimens, cell lines, and xenografts to discover the mechanism(s) behind this transformation, invasiveness, and metastasis are in progress. 

Alteration of cell cycle proteins (e.g., cyclin D1, pRb, and p16) has a role in transformation and progression in melanocytic tumors. It has been shown that progressive loss of p16 can be seen in transformation of benign nevi to melanoma and to metastatic melanoma. Progressive increase in expression of cyclin D1 and pRb is associated with progression to melanoma cells; however, cyclin D1 and pRb show relative decrease in thick melanoma and metastatic melanoma [81].

Higher expression of PAR-1 (protease-activated receptor-1) is seen in melanoma cell lines and tissue specimens. Upregulation of PAR-1 mediates high levels of Cx-43 (gap junctional intracellular communication molecule connexin) expression. This molecule is involved in tumor cell diapedesis and attachment to endothelial cells [82]. Type I collagenase and PAR-1 activating functions of MMP-1 (matrix metalloproteinase-1) are required for melanoma progression. Highly expressed MMP-1 is suggested to be involved in progression of noninvasive melanoma to invasive vertical growth phase by degrading type I collagen of skin [83]. 

Protein Kinase C (PKC) mediates signals for cell growth and is a target of tumor-promoting phorbol esters in malignant transformation [84]. 

Downregulation of E-cadherin and upregulation of N-cadherin may be seen in melanoma cells. Such shift of cadherin profile may have a role in uncontrolled proliferation, invasion, and migration [85]. 

S100A1, S100B, Bcl-2, and CD44 have been described in transformation of melanocytes to melanoma cells. S100A1 expression is increased in contrast to S100B, which shows higher expression in benign nevi. The Sviatoha et al. demonstrated studies of a higher expression of CD44 antigen in melanomas with known metastases than in those without metastases, but this difference was not statistically significant [86]. Interaction of the transcription factor E2F-1 with RGFR can act as driving force in melanoma progression [87]. 

The studies of Mehnert et al., based on the fact that angiogenesis is one of the factors required for progression and melanoma metastasis, demonstrated that vascular endothelial growth factor (VEGF) and its receptors (VEGF-R1, VEGF-R2, and VEGF-R3) are higher in melanomas and advanced melanomas than in benign nevi. VEGF-R2 shows higher expression of VEGF-R2 in metastatic melanomas than in primary melanoma [88]. 

Using immunochemistry on human tissue, it has been shown that there is significantly higher cortactin (a multidomain actin-binding protein important for the function of cytoskeleton) expression in melanomas than in nevi and higher expression in metastatic melanoma than in invasive primary melanomas [89]. MHC (major histocompatibility complex) molecule overexpression in earlier stages of melanoma and downregulation in metastatic malignant melanoma have been observed [90]. 

PEDF (pigment epithelium-derived factor) loss appears to be associated with invasive phenotype and malignant progression [91]. Deregulation of microRNAs (miRNAs) using cell lines from primary or metastatic melanoma contributes in formation and progression of melanoma [92]. Melanoma chondroitin sulfate proteoglycan (MCSP) facilitates the growth, motility, and invasiveness of tumor cells. MCSP expression is associated with increased expression of c-Met and HGF. c-Met inhibition limits growth and motility of melanoma cell lines [93]. Up-regulated expression of C-Raf is seen in a subset of melanomas [94]. 

ATP-binding cassette (ABC) transporters regulate the transport of physiologic substrates. ABC-transporter mRNA expression profile may have some roles in melanoma tumorigenesis [95]. It has been suggested that there is transient upregulation of cDNA clone pCMa1 in neoplastic progression of melanocytes [96]. 

It has been shown that angiogenesis and metastasis can be inhibited by heparin and its derivatives. The study conducted by Kenessey et al. revealed that fragments of heparin, not involved in its haemostatic effect, may have a role in antimigratory and antimetastatic processes [97]. 

## 10. Treatment of Malignant Melanoma and Followup

Avoiding sunlight if possible, frequent use of sunscreen and routine checkups in high risk patients are important preventive measures. Sometimes the use of sunscreen is associated with higher incidence of malignant melanoma but in fact this is in due to modified sun-exposure behavior [98, 99]. Adequate clear resection margins handling of the reexcision specimens and sentinel lymph node biopsies are important factors in management of malignant melanoma. A variety of protocols for excision of primary cutaneous melanomas exist. One commonly used protocol [100–104] is in situ melanoma: 0.5 cm clear margin, <1 mm: 1 cm clear margin 1–2 mm 1–2 cm clear margin (depending on the location), 2–4 mm 2 cm clear margin, >4 mm 3 cm clear margin. The sentinel lymph node is the first node in the basin of the regional lymph node that picks up the ^99m^Tc and/or blue dye. Sentinel node biopsy has had a significant impact on managing patients of melanoma [105]. 

Surgical excision, interferon therapy, hypothermic isolated limb perfusion with melphalan, CO2 laser ablation, and intralesional BCG have been used for treatment of in-transit melanoma. In-transit melanoma metastasis is defined by in-transit cutaneous malignant melanoma deposits between the site of excision and the draining lymph nodes [106] more than 2 cm from primary melanoma, which is different from satellite metastasis defined by lesions less than 2 cm from the primary melanoma. Despite of different definitions the biologic behaviour of both is similar and is categorized as intralymphatic metastasis as another criterion in the N category regardless of the number of lesions based on the final version of the 2009 AJCC melanoma staging and classification [107, 108]. Patients with positive nodes or node-negative melanomas thicker than 4 mm, ulceration, or Clark's level IV or V may benefit of adjuvant therapy. Interferon-alpha 2b is the most commonly used FDA-approved adjuvant therapy [109]. There is no definite proof that longevity of patients is affected by routine laboratory tests such as lactate dehydrogenase (LDH) and/or imaging studies such as CT scan, MRI, and PET scan. There are several guidelines, which recommend limited use of laboratory test and imaging based on the disease stage. Low yield, high rate of false-positive tests, and lack of significant impact of early detection of metastases on survival argue that chest X-ray and serum LDH should probably not be accepted into routine clinical practice in clinically localized melanoma in the absence of data supporting their use [110, 111]. However, patients with higher stage may benefit from these tests. 

## 11. Final Version of 2009 AJCC Melanoma Staging and Classification

In the final version of 2009 AJCC, the 7th edition [107], the mitotic rate per mm2 has been added to staging of melanoma. Mitosis = 1 per mm^2^ is included in primary criterion for defining T1b melanoma. Immunohistochemical detection of nodal metastasis has also been incorporated and must include at least one melanoma-associated marker (e.g., HMB45, Melan-A, and Mart-1) unless diagnostic cellular morphology is present. In addition there is no lower threshold of staging N disease [107]. The new Melanoma Staging Database clearly demonstrates that an elevated LDH is an independent and highly significant predictor of survival or outcome of stage IV malignant melanoma. LDH is amongst the most predictive independent factors of diminished survival when is analyzed in a multivariate analysis [107, 112, 113].
